# Supplementary material for: Scorpion (Hottentotta tamulus) venom pre-exposure delays functional recovery in mice following peripheral nerve injury
Source: PLoS One. 2025 Aug 19;20(8):e0330600. doi: 10.1371/journal.pone.0330600 (PMC12364329; doi:10.1371/journal.pone.0330600)
Supplement: S1 File — (DOCX) [file pone.0330600.s001.docx]

**Supplementary Figures:**


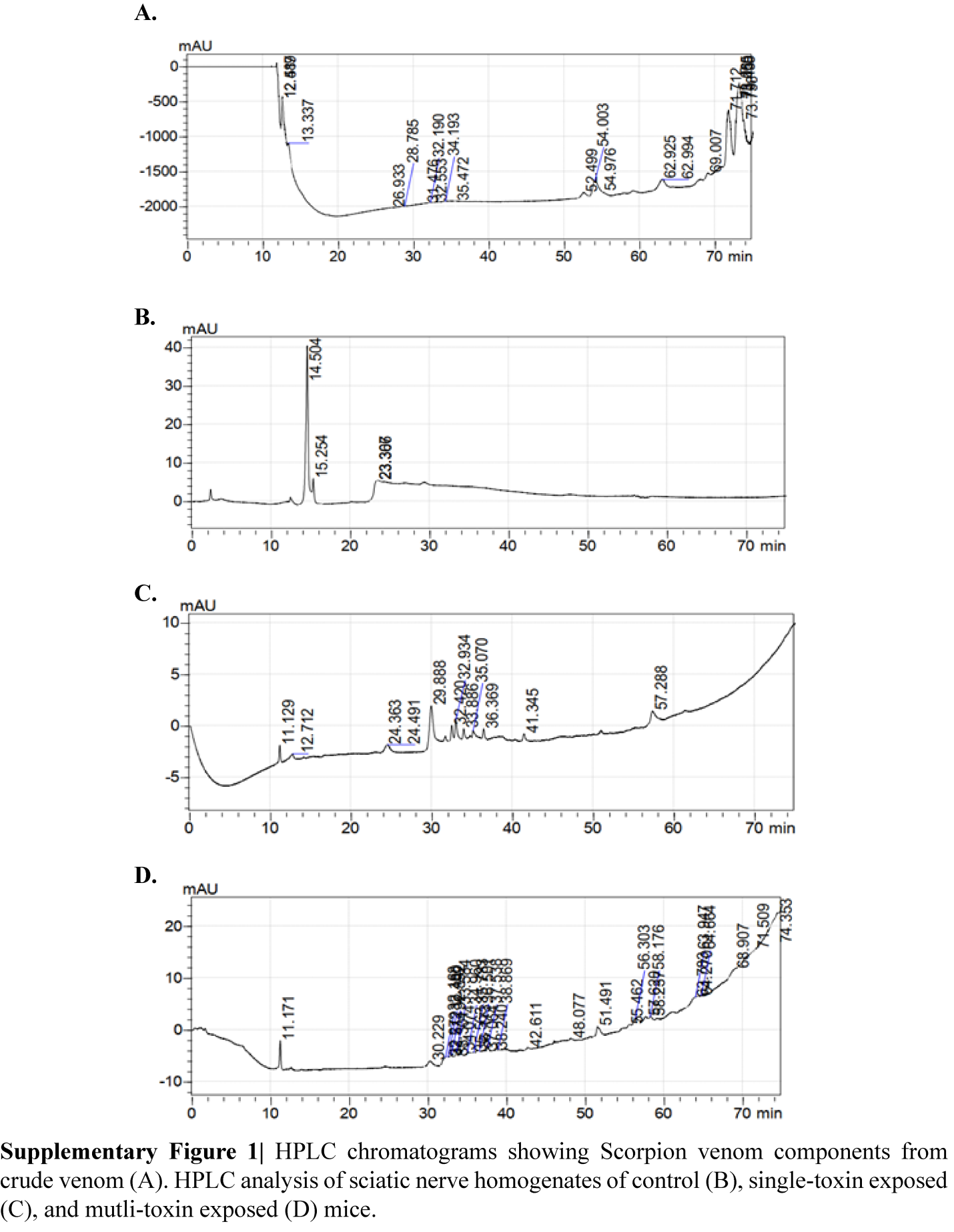


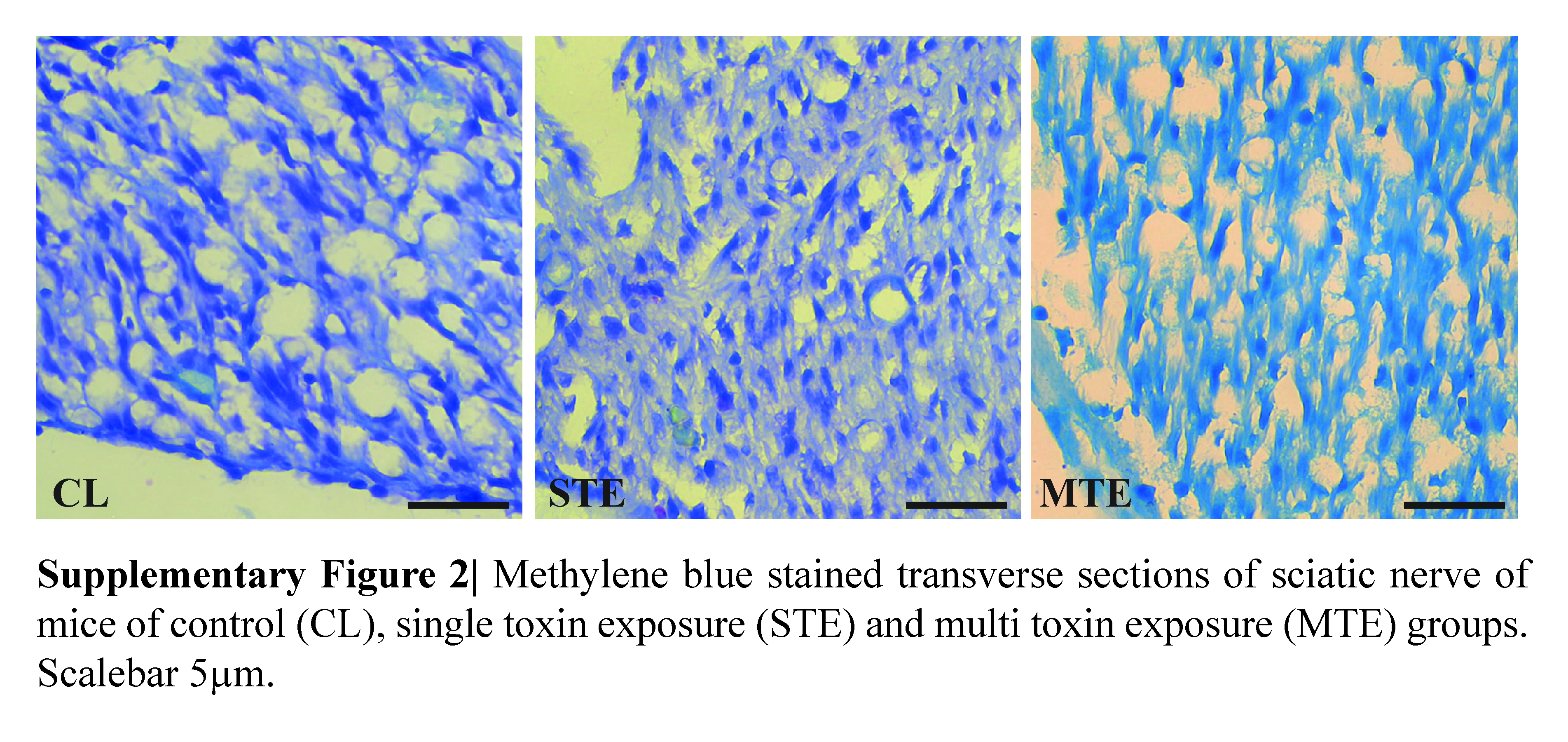


**Humane Endpoints:**

The humane endpoints for the study are mentioned at appropriate sections of the article. Briefly, a growing and well-established data on mice peripheral (sciatic) nerve regeneration following crush injury unveils the complete regain of sensori-motor functional recovery in 4-weeks time [[1](#_ENREF_1), [2](#_ENREF_2)]. Similarly, extent of regeneration of injured sciatic nerve and neuromuscular junction restoration in mice gastrocnemius muscles is reported at 14 days post sciatic nerve crush injury[[3](#_ENREF_3), [4](#_ENREF_4)]. Hence humane endpoints of the presented study were selected appropriately.

1. Au, N.P., et al., *Ciguatoxin reduces regenerative capacity of axotomized peripheral neurons and delays functional recovery in pre-exposed mice after peripheral nerve injury.* Sci Rep, 2016. **6**: p. 26809.

2. Ma, C.H., et al., *Accelerating axonal growth promotes motor recovery after peripheral nerve injury in mice.* J Clin Invest, 2011. **121**(11): p. 4332-47.

3. Ramli, D., et al., *The Changes in Rats with Sciatic Nerve Crush Injury Supplemented with Evening Primrose Oil: Behavioural, Morphologic, and Morphometric Analysis.* Evid Based Complement Alternat Med, 2017. **2017**: p. 3476407.

4. Zhu, H., et al., *EZH2-dependent myelination following sciatic nerve injury.* Neural Regen Res, 2025. **20**(8): p. 2382-2394.
