# Supplementary material for: Scorpion (Hottentotta tamulus) venom pre-exposure delays functional recovery in mice following peripheral nerve injury
Source: PLoS One. 2025 Aug 19;20(8):e0330600. doi: 10.1371/journal.pone.0330600 (PMC12364329; doi:10.1371/journal.pone.0330600)
Supplement: Attachment — Submitted filename: Response to Editor.docx [file pone.0330600.s002.docx]

**Response to Editor Comments:**

**Methods Section：**

**Comment:** The authors administered 1 mg/kg of the toxin intraperitoneally to Swiss albino mice. It is necessary to not only cite references but also explicitly explain the rationale behind this dosage.

**Response:** We agree with the editor to acknowledge the reference of dose selection (1mg/kg). Following statements are added in the methodology section of “Swiss albino mice grouping and dosing”

Lyophilized scorpion venom dose of 1mg/kg (in 5%DMSO containing distilled water) was selected for intraperitoneal administration based on previous study. The selected dose was safe for Swiss albino mice and caused no observable adverse effects on mice health during study duration [26].

**Comment:** Additionally, the reason for administering MTE once per week for four consecutive weeks should be clearly stated. Why was the treatment repeated exactly four times?

**Response:** We thank the editor for insightful comment on the rationale of multiple dosing. The following paragraph is added in the discussion section to partially address the comment.

In real world, scorpion envenomation at daily basis is not reported however, frequent scorpion sting incidences are reported over time. In current study, the aspect of repeated scorpion envenomation (in MTE group) was considered equally important along with single envenomation (STE mice). Weekly administration of venom safe dose (1mg/kg) was hypothesized to mimic the real-life envenomation risks. Of note, a bi-weekly administration of venom is reported to cause high mortality , and excessive exposure of sub-lethal doses (0.01mg/kg and 0.02mg/kg) caused painful effects with frequent numbness signs in toxin injected paw [56]. Prolong exposure of toxin (exceeding 4-weeks duration) might lead to excessive functional adaptation and is less likely to occur in habitats. Importantly, venom components influence the activity of critical ion channels (such as Na+ and K+ ion channels) and frequent envenomation over longer duration of time could lead to neurotoxicity and cardiovascular collapse leading to animal mortality. Therefore, in current study a modest dose administration schedule once a week for 4 consecutive weeks was selected to maintain a consistent toxic burden, to estimate cumulative influence on nerve regeneration.

**Comment:** In the Mice Surgery for Sciatic Nerve Crush procedure, what was the rationale for covering the crushed site with muscle tissue after the injury? Please provide a clear explanation.

**Response:** The anatomical presence of sciatic nerve in mice is below the overlying muscles. In performing sciatic nerve crush injury, the overlying muscles are slightly displaced to observe the sciatic nerve and then crush injury is performed. Following sciatic nerve crush injury, it is covered by restoring the position of overlying muscles. In submitted article, the statement “The nerve was covered with muscles and skin was sutured with 5-0 silk-derived sutures (B.T. Sutures)” describes the same. However, if it confuses the readers, we can amicably revise the statement.

**Results Section：**

**Comment:** The Methylene blue staining images of regenerating sciatic nerve (SN) sections appear to be of poor quality. This makes quantitative analysis difficult. Why were the images not presented in color?

**Response:** Appropriate changes are made and the methylene blue stained histology pictures are shown.

**Comment:** A detailed explanation of how the quantification was specifically performed using ImageJ is required.

**Response:** A detailed explanation of quantification using ImageJ is described in Supplemental file.

**Comment:** The GCM staining in Figure 2C also has quality issues. The staining sites are unclear, making interpretation difficult. Fluorescent immunostaining using a marker for axonal expression would be preferable. Alternatively, higher magnification images with enhanced contrast should be provided.

**Response:** The Figure 2C is revised as per instructions of the editor.

**Comment:** Furthermore, it would be beneficial to present additional stained images, even as Supplementary Data. The current data are insufficient to substantiate the authors' hypothesis.

**Response:** Supplemental figures 1 and 2 are added amicably.

1.	Butt SA, Tahir HM, Ali S, Tariq M, Hassan A, Summer M, et al. Evaluation of anti-scorpion (Hottentota tamulus) venom potential of native plants extracts using mice model. Toxin Reviews. 2022;41(3):806-16: doi: PubMed PMID: PMID: PubmMed PMCID:

2.	Thiruchelvam M, Brockel B, Richfield EK, Baggs RB, Cory-Slechta DA. Potentiated and preferential effects of combined paraquat and maneb on nigrostriatal dopamine systems: environmental risk factors for Parkinson’s disease? Brain research. 2000;873(2):225-34: doi: PubMed PMID: PMID: PubmMed PMCID:

3.	Gunas V, Maievskyi O, Raksha N, Vovk T, Savchuk O, Shchypanskyi S, et al. Study of the Acute Toxicity of Scorpion Leiurus macroctenus Venom in Rats. The Scientific World Journal. 2024;2024(1):9746092: doi: PubMed PMID: PMID: PubmMed PMCID:
