# Supplementary material for: Scorpion (Hottentotta tamulus) venom pre-exposure delays functional recovery in mice following peripheral nerve injury
Source: PLoS One. 2025 Aug 19;20(8):e0330600. doi: 10.1371/journal.pone.0330600 (PMC12364329; doi:10.1371/journal.pone.0330600)
Supplement: Attachment — Submitted filename: Response to Reviewers_20250617-1.docx [file pone.0330600.s003.docx]

**Response to Reviewers**

We are thankful to the reviewers for insightful comments on the research article entitled “Scorpion (*Hottentotta tamulus*) venom pre-exposure delays functional recovery in mice following peripheral nerve injury” submitted for publication to the PlosOne Journal.

The article is revised in light of the suggestions and comments of the reviewers, details of which are given below,

**Reviewer 1:**

**General Comment:** Khan and colleagues studied the effects of the scorpion *Hottentotta tamulus* venom in peripheral nerves in mice. They showed that the venom persists following injection and that compromises the recovery of nerves. The study is comprehensive and the results support the hypothesis. Specific comments are listed below.

**General Response:** We are encouraged to revise our manuscript by the very valid comments of the reviewer 1. Point-to-point comments of the reviewer are addressed below and appropriate changes are made in revised manuscript.

**Comment:** Is there any previous evidence of scorpion venoms (from any specie) persistence in the nervous systems (as authors describe for ciguatoxins)?

**Response:** currently, to our knowledge, there is no direct evidence of persistence of scorpion venom components in the nervous system for extended periods in the same manner as ciguatoxins, which are known to integrate into neural membranes and exert long-term effects (Au et al., 2016). However, several studies support the idea of functional persistence of scorpion venom effects on the nervous system for hours to few days, primarily due to slow clearance of certain neurotoxic peptides and their prolonged impact on ion channels and neuroimmune responses.

*Tityus serrulatus* venom was observed for neuro-excitatory and inflammatory responses in rodent models with some physiological and behavioral changes for up to 72 hours post exposure. However, no molecular retention was demonstrated in these studies, while suggesting a sustained downstream effect of venom exposure on neuronal function (Pessini et al., 2003, Nencioni et al., 2000, Van Fraga et al., 2015).

Potassium and sodium channels were highly expressed, aligning with scorpion neurotoxicity profiles. This support the idea of neurotoxic activity post-envenomation, however, structural persistence of scorpion venom components is unproven in contrast to ciguatoxin. Additionally, metalloproteinase and other bioactive peptides may contribute to tissue inflammation and prolonged biological effects reinforcing the observations of functional persistence in the nerve regeneration and gene expression studies (Costal-Oliveira et al., 2015).

**Comment:** Why was the intraperitoneal route chosen for this studies since most envenomations in human occur via subcutaneous or intramuscular routes?

**Response:** The intraperitoneal (IP) route was selected for the current study due to its effectiveness in ensuring rapid and systemic distribution of venom in the small animal model selected in the current study (Swiss albino mice). IP route is utilized because it allows the controlled delivery, reduced local tissue variability and reproducibility in dose-response outcomes in venom toxicology research. The IP route was chosen to enable consistent exposure across experimental groups and to better mimic the systemic effects of venom in controlled conditions.

Intraperitoneal injection of *T. serrulatus* and *T. bahiensis* scorpions induced electrographic and behavioral alterations, in previous studies, suggesting intraperitoneal route as a potential route of venom administration in pre-clinical studies (page no. 236, 3^rd^ paragraph from (Nencioni et al., 2009)).

**Comment:** Was the expression of Gapdh similar in all groups? This is important since this gene was used for normalization.

**Response:** Yes, Gapdh expression was consistent across all experimental groups, as validated by quantitative polymerase chain reaction (qPCR) analysis by observing the Ct values. Gapdh showed least variation across control (CL), single toxin exposure (STE) and multiple toxin exposure (MTE) groups hence served as an internal control for normalizing expression levels of selected genes in the current study.

**Comment:** Page 3, line 53: please correct “present” to “be present”.

**Response:** Appropriate change is made in line with the reviewer’s comment.

**Comment:** Page 5: define PNS.

**Response:** Appropriate change is made in revised manuscript.

**Comment:** Page 8: correct “1.5μL” to 1.5mL

**Response:** Correction is made in revised manuscript.

**Comment:** Page 14: correct “oneway” to “one-way”

**Response:** Correction is made in the revised manuscript.

**Reviewer 2:**

**General Comment:** Based on behavioral testing, it was discovered that scorpion venom delayed neural recovery, and the author also explored the expression levels of related effect genes. Below are the questions the author needs to address:

**General Response:** We appreciate the comments of the reviewer 2 to improve the manuscript submitted for publication in the PlosOne journal. The manuscript is revised and the comments of the reviewer 2 are addressed below,

**Comment:** The HPLC results confirmed the presence of scorpion venom residues, but did not provide an accurate material analysis of the venom. Additionally, what are the characteristic components of scorpion venom used for detecting residues? This needs to be clarified.

**Response:** We acknowledge the limitation regarding the material characterization. The current study was designed to detect the residual components of scorpion venom based on established protocols. However, full molecular characterization was not conducted in the current work, the retained peaks match previously described low molecular weight toxins (6-8kDa) are possibly associated with neurotoxic effects. We are thankful for the reviewer to highlight this important point, however the characterization of scorpion venom components was not the objective of the research, as one of PhD scholar’s PhD research project involves the LC-MS based characterization of the scorpion venom and hence it was decided not to go for venom component characterization in current study. Further, we have mentioned this limitation in last paragraph of the discussion sections (line 504). However, we feel the suggested comment needs to be addressed through a separate study following ethical and departmental approvals. And the identified component of scorpion venom persisted in sciatic nerves might be explored for its regeneration-modulating effects through a separate *in vivo* study.

**Comment:** The author conducted tests on the expression levels of related effect genes. Are there any other image-based supporting evidences (such as fluorescence, histochemistry, etc.) available to corroborate these findings?

**Response:** We are thankful to the reviewer for highlighting an important point of validating qPCR data on protein level through immune-histochemistry. In current study, in addition to the gene expression data, histological analysis of the sciatic nerve tissue was performed using methylene blue staining in an attempt to observe the myelination around regenerating axons to correlate the compromised expression of regeneration-associated genes with the sub-optimal myelination in nerves of intoxicated mice. However, lack of fluorescence microscope, antibodies and relevant logistics restrained us to perform the mentioned fluorescence based detection of protein products of the regeneration-associated genes (Gap43, Atf3, Sox11 etc). Therefore, we regret to have such relevant data in current article.

In the manuscript, last paragraph of discussions section reports the limitation of the current study and in line with the reviewer’s comment, the limitation of unavailability of Western blot equipment and facilities are mentioned (Line 510 of the manuscript) which restrained us to explore the expression levels of regeneration-associated genes at protein levels.

**Comment:** Some spelling errors, such as "N represent" at line 225 of the R1 revision.

**Response:** “N” represent Normal paw (Contralateral- uninjured) foot-prints. Appropriate revision is made in revised version.

AU, N. P., KUMAR, G., ASTHANA, P., TIN, C., MAK, Y. L., CHAN, L. L., LAM, P. K. & MA, C. H. 2016. Ciguatoxin reduces regenerative capacity of axotomized peripheral neurons and delays functional recovery in pre-exposed mice after peripheral nerve injury. *Sci Rep,* 6**,** 26809.

COSTAL-OLIVEIRA, F., GUERRA-DUARTE, C., CASTRO, K. L., TINTAYA, B., BONILLA, C., SILVA, W., YARLEQUE, A., FUJIWARA, R., MELO, M. M. & CHAVEZ-OLORTEGUI, C. 2015. Serological, biochemical and enzymatic alterations in rodents after experimental envenomation with Hadruroides lunatus scorpion venom. *Toxicon,* 103**,** 129-34.

NENCIONI, A. L. A., CARVALHO, F. F., LEBRUN, I., DORCE, V. A. C. A. & SANDOVAL, M. R. L. 2000. Neurotoxic effects of three fractions isolated from Tityus serrulatus scorpion venom. *Pharmacology & toxicology,* 86**,** 149-155.

NENCIONI, A. L. A., LOURENCO, G. A., LEBRUN, I., FLORIO, J. C. A. & DORCE, V. A. 2009. Central effects of Tityus serrulatus and Tityus bahiensis scorpion venoms after intraperitoneal injection in rats. *Neuroscience letters,* 463**,** 234-238.

PESSINI, A. C., DE SOUZA, A. M., FACCIOLI, L. H., GREGóRIO, Z. M. & ARANTES, E. C. 2003. Time course of acute-phase response induced by Tityus serrulatus venom and TsTX-I in mice. *International immunopharmacology,* 3**,** 765-774.

VAN FRAGA, I. T., LIMBORCO-FILHO, M., LIMA, O. C. O., LACERDA-QUEIROZ, N., GUIDINE, P. A. M., MORAES, M. F. D., ARAúJO, R. N., MORAES-SANTOS, T., MASSENSINI, A. R. & ARANTES, R. M. E. 2015. Effects of tityustoxin on cerebral inflammatory response in young rats. *Neuroscience Letters,* 588**,** 24-28.
